# Retinal vein occlusion with cerebral infarction in a preterm neonate: a case report

**DOI:** 10.1186/s12887-021-02989-2

**Published:** 2021-11-16

**Authors:** Xiuyu Zhu, Xiaojing Cai, Xiaohong Zhou, Yian Li, Chenhao Yang

**Affiliations:** Department of Ophthalmology, Children’s Hospital of Fudan University, National Children’s Medical Center, Wanyuan Road No.399, Shanghai, 201102 China

**Keywords:** Retinal vein occlusion, Newborn, Stroke

## Abstract

**Background:**

Retinal vein occlusion (RVO) is a common disease that causes blindness in elderly patients, and cerebral infarction is also a severe disorder impairing the health of individuals. Both diseases are not common in neonates and are related to thrombosis. To date, only one case of simultaneous occurrence of RVO with intracranial haemorrhage in a full-term neonate has been reported.

**Case presentation:**

A preterm neonate was diagnosed with cerebral infarction and RVO. Retinal haemorrhage and macular oedema were detected in the left eye after the onset of ipsilateral stroke. Although the retinal conditions in this case resolved spontaneously without ocular treatment, the long-term effect on visual function is still unknown.

**Conclusions:**

Given that ocular fundus examinations are rarely performed in paediatric stroke patients, a screening fundus examination in these newborns with stroke might be worth considering.

## Background

Retinal vein occlusion (RVO) is the second leading cause of retinal vascular blindness after diabetic retinopathy. Vision loss is frequently associated with macular oedema (ME) and neovascularization with secondary vitreous haemorrhage and/or neovascular glaucoma. RVO is common in middle-aged and older adults but rare in children since its prevalence increases with advanced age [1]. Systemic risk factors, including hypertension, diabetes mellitus, and hyperlipidaemia, are commonly related to RVO, and they can also occur secondary to thrombophilia and other processes, such as inflammation, vasospasm, or compression [2]. RVO is clinically diagnosed based on the funduscopic findings of dilated tortuous retinal veins with flame haemorrhages, dot and blot haemorrhages, and/or cotton wool spots. Optical coherence tomography (OCT) is helpful in confirming the presence of ME, and fluorescein angiography (FA) can facilitate the visualization of peripheral capillary nonperfusion and macular ischaemia and the detection of clinically subtle neovascularization [2]. According to the site of obstruction, RVO can be divided into central retinal vein occlusion (CRVO), branch retinal vein occlusion (BRVO) and hemiretinal vein occlusion (HRVO). HRVO is an obstruction involving the anterior part of a trunk of the central retinal vein and can be considered a separate entity or a subtype of either CRVO or BRVO [3]. Neonatal cerebral infarction is an uncommon condition associated with embolic, thrombotic or other events, such as perinatal asphyxia, ischaemia, and trauma [4, 5].

Most patients may present with the symptoms of seizures, hypotonia and lethargy [5].

The relationship between RVO and stroke is controversial; although some studies indicated that they were risk factors for each other [6, 7], simultaneous occurrence is rare. We report the case of a premature infant with acute cerebral infarction and subsequent unilateral HRVO. To our knowledge, no case of RVO in preterm newborns or simultaneous RVO with stroke has been reported in the literature. This case report can broaden the age spectrum of RVO and provide insights into the benefits of ophthalmologic fundus examination for neonatal stroke.

## Case presentation

A female infant was delivered by caesarean section due to placenta previa at 35 weeks gestation to a Chinese 33-year-old woman (gravida 1, para 1) with a pelvic operation history. The pregnancy was a result of in vitro fertilization with the parents’ own gametes. Antenatal screening and scans were normal. The birth weight was 2670 g. The Apgar scores were 9 and 10 at 1 and 5 min, respectively, after birth. The infant was transported to the neonatology ward for observation, and she showed few abnormal symptoms other than mild dysphagia. Vitamin K was routinely used for preventing bleeding.

**Fig. 1:**
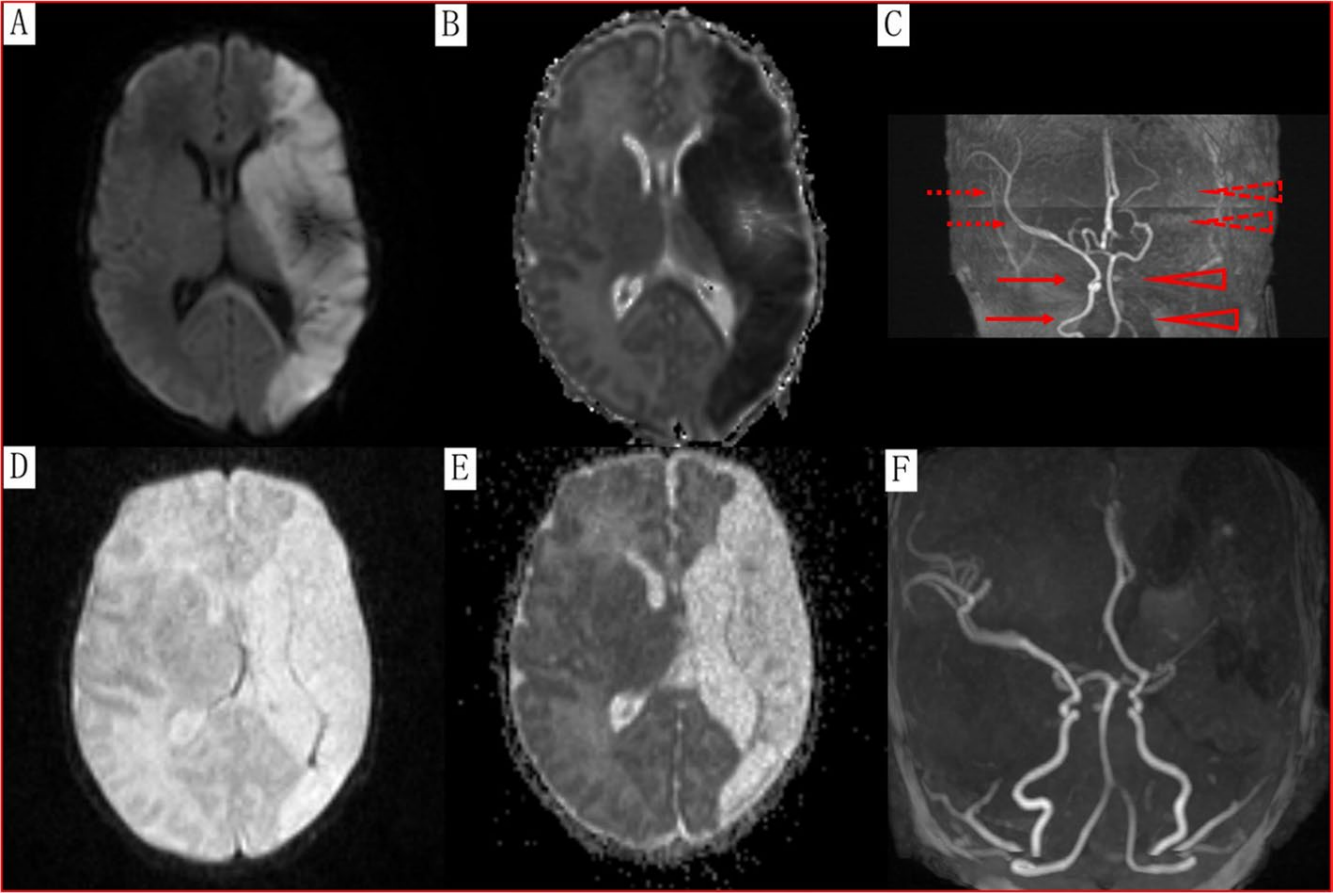
Brain MRI and MRA. Seven days after birth (**A**&**B**&**C**). Diffusion-weighted (**A**) and apparent diffusion coefficient (**B**) MRI, respectively revealed hyperintense and hypointense signals in the left hemisphere, indicating complete left middle cerebral artery (MCA) infarction. **C** MRA showed absent flow signal in the expected location of the left internal carotid artery (ICA) and MCA due to occlusion (red solid and dotted arrowheads indicate expected course of left ICA and MCA as compared to red solid and dotted arrows that indicate course of right ICA and MCA). Thirty days after birth (D&E&F). Diffusion-weighted (**D**) and apparent diffusion coefficient (**E**) MRI showed a slightly hypointense and a hyperintense signal in the left MCA territory, respectively. **F** MRA showed reperfusion of the left ICA and MCA, and the MCA was obviously stenotic with fewer branches

Six days after birth, brain ultrasonography screening revealed abnormally enhanced echogenicity in the left hemisphere. Then, the newborn was transferred to the neonatal intensive care unit with a diagnosis of acute stroke. On day 7, brain magnetic resonance imaging (MRI) showed left middle cerebral artery (MCA) occlusion with intracranial venous engorgement, and magnetic resonance angiography (MRA) showed an absent flow signal of the left internal carotid artery (ICA) and MCA (Fig. 1). The laboratory results of coagulopathy were as follows: antithrombin III was 65%, antithrombin III antigen was 14.8 mg/dl, protein C was 33% and protein S was 39.2%. The abnormal blood coagulation function indices included D-dimer (DD, 7.1, normal range, 0–0.5 mg/ml), activated partial prothrombin time (APTT, 42.0, normal range, 26–440 s), and fibrinogen degradation product (FDP, 29.71, normal range, 0–5 μg/ml). The abnormal thromboelastogram parameter was reaction time (R), 3.2 min (normal range, 5–110 min). There were no clinical neurologic symptoms or signs to be found except for mild dysphagia. Low molecular weight heparin calcium was used for antithrombotic therapy.

**Fig. 2:**
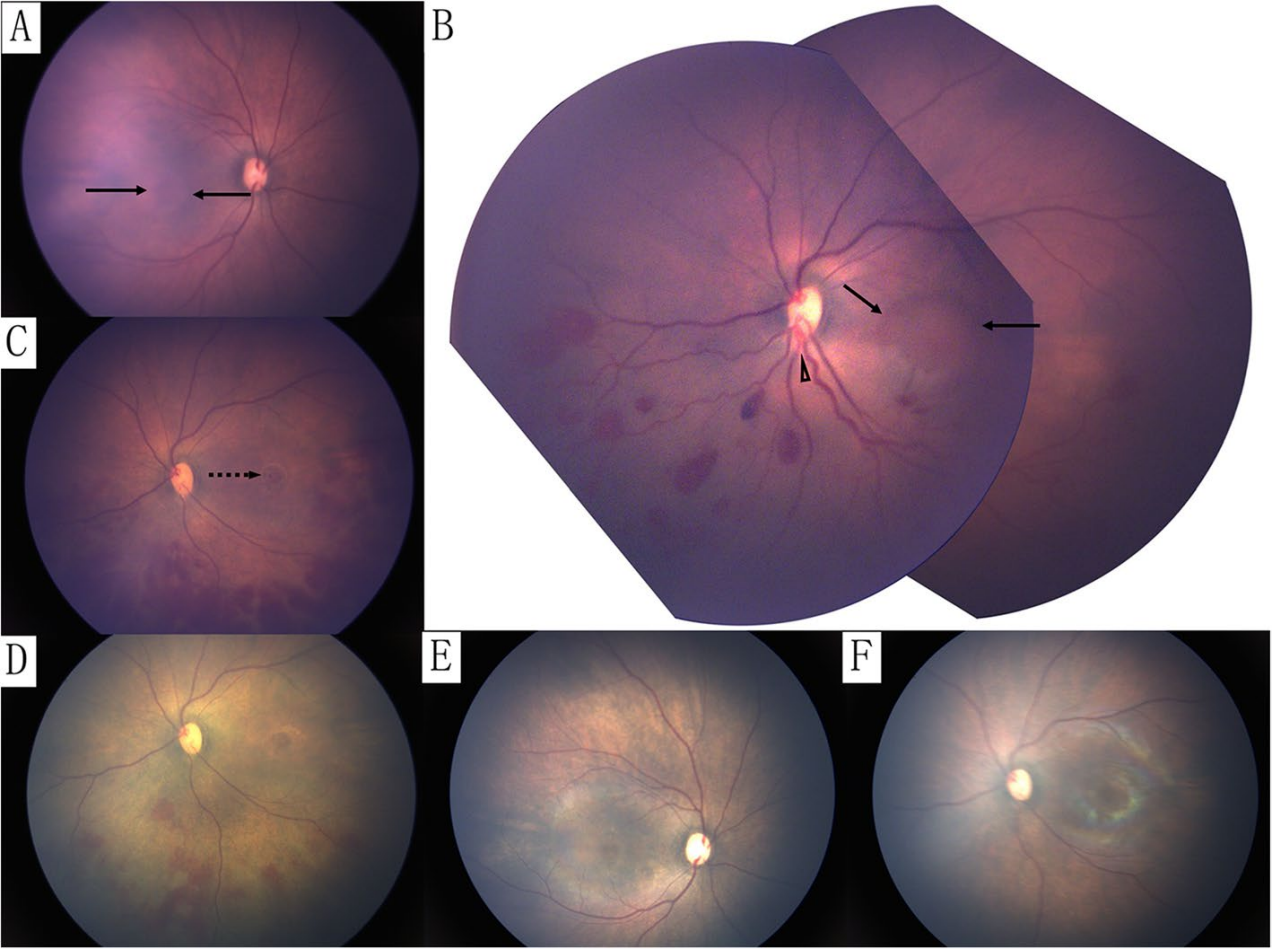
RetCam fundus photograph. Eight days after birth (**A**&**B**). Bilateral macular oedema (ME) in right (**A**) and left (**B**) eyes (arrow). **B** The left fundus photo shows inferior retinal haemorrhages (RHs) and tortuous dilated veins with proximal stenosis (arrowhead) in the location of the optic disc rim. **C** Increased RHs with submacular haemorrhage (dotted arrow) in the left eye at 15 days after birth. Note that the inferior retinal vein became stenotic in comparison with the corresponding superior vessel. **D** Most RHs resolved in the left eye at 22 days. Fifty-one days after birth (**E**&**F**). ME in right (**E**) and left (**F**) eyes, and all RHs had resolved in the left eye (**F**)

**Fig. 3:**
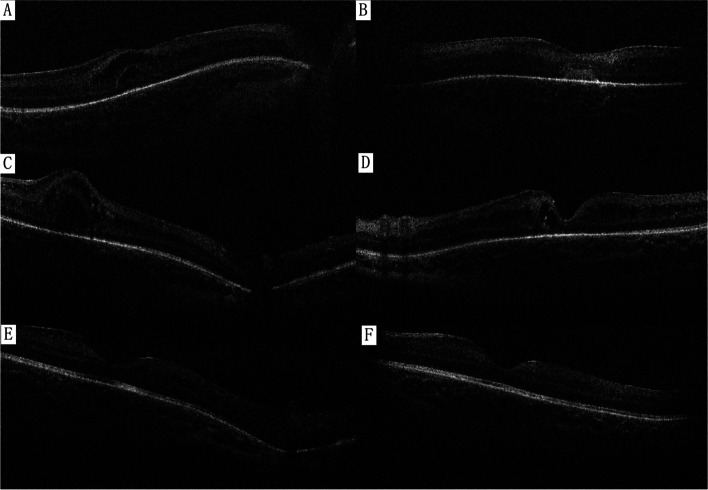
OCT at day 22 (**A**&**B**), day 37 (**C**&**D**), and 3 months (**E**&**F**). **A**&**B** Cystoid macular oedema (ME) in the right (**A**) and left eyes (**B**) with subretinal haemorrhage (SRH). **C**&**D** ME in the right (**C**) and left eyes (**D**) showed that SRH had resolved in the left eye. **E**&**F** ME and SRF resolved in right (**E**) and left eye (**F**)

On day 8, the screening fundus photograph (RetCam III, Clarity Medical Systems, USA) showed ME in both eyes and sporadic intraretinal haemorrhages along the inferior retinal vessels in the left eye, and the inferior retinal vein was dilated and tortuous (Fig. 2). FA was not performed because of the poor systemic condition for general anaesthesia. Unilateral HRVO and bilateral ME were diagnosed, and observation for retinal diseases was adopted in consideration of no obvious retinal or choroidal neovascularization being found. The fundus photograph at day 15 showed new submacular haemorrhage and more intraretinal haemorrhages in the inferior retina of the left eye, but the retinal vein restored to a normal pattern (Fig. 2). On day 22, a fundus photo showed that most intraretinal haemorrhages had resolved. OCT (Leica/Bioptigen Envisu C2300, RTP, NC) confirmed bilateral ME and submacular haemorrhage in the left eye (Fig. 3).

On day 30, the cranial MRI showed cerebral atrophy as a sequela of left MCA occlusion and an increased volume of the left ventricle. MRA showed reperfusion of the left ICA and MCA (Fig. 1).

Since the patient’s condition was stable, the child was discharged at the age of 31 days. The submacular haemorrhage had resolved at day 37 (Fig. 3), and intraretinal haemorrhages had resolved at day 51. Bilateral ME had resolved at 3 months (Fig. 3).

## Discussion and conclusions

The incidence of arterial ischaemic stroke during the neonatal period is approximately 1 in 2700 live births [8]. There are many risk factors or medical conditions associated with perinatal arterial ischaemic stroke. However, in this case, we did not detect any obvious cause after a comprehensive general examination, including hypercoagulable states, urine gas chromatography-mass spectrometry study, serum amino acid analysis, echocardiography, gene test, etc.

Retinal haemorrhage (RH) is common in newborns, and the differential diagnosis usually includes birth-related RH, abusive head trauma (AHT) and increased intracranial pressure (ICP). Birth-related RHs are frequently bilateral, intraretinal and in the posterior pole [9]. AHT is a neurological injury caused by shaking, inflicted blunt impact or a combination of both [10]. RHs in AHT are frequently abundant, multilayered, and scattered in the majority of the retina. Although raised ICP is an isolated cause for RH in paediatric patients, it is rare to detect RH in children with nontraumatic elevated ICP [11]. When present, RHs are mostly intraretinal and limited to the peripapillary region with the presence of papilledema [11, 12]. In this case, the child was delivered by caesarean section without a history of blunt trauma. The RHs were unilateral and distributed along the vessels in the inferior retina with dilated and tortuous retinal veins, which is common in RVO but rare in the aforementioned three diseases.

The pathogenesis of RVO is different between elderly and young patients. Hypertension, hypercholesterolemia and status after cerebral infarction/haemorrhage are considered the major risk factors for RVO in elderly patients [6]. In contrast, procoagulant states predominate the mechanisms of RVO in young patients [13]. In this case, RVO is likely to have resulted from hypercoagulable states after stroke. Although the association between venous and arterial thrombosis remains unclear, venous thromboembolism, usually deep vein thrombosis (DVT), is a common complication following acute stroke. During the first fortnight after stroke without prophylactic therapy, the incidence of DVT varies between 27 and 75% [14]. Likewise, patients with previous cerebral infarction or haemorrhage had a higher risk of subsequent RVO [6]. However, to our knowledge, only one case of a full-term neonate with simultaneous RVO and intracranial haemorrhage has been reported [15]. Our case is the first preterm newborn with cerebral infarction and simultaneous RVO.

Cystoid ME due to RVO can lead to significant visual loss. However, it can also frequently present in the inner nuclear layer of premature infants, especially at 37 weeks postmenstrual age. These cystoid changes might be due to a remodelling of the foveal architecture or, perhaps, elevated vascular endothelial growth factor levels [16]. In this case, the presentation of bilateral ME suggested that ME was probably due to premature birth rather than RVO, and the spontaneous regression of ME confirmed our assumption.

Subretinal haemorrhage (SRH) in the left eye was an interesting finding. There are only a few reports of SRH in newborns with ROP [17, 18], AHT [19] or infection [20]. Although SRH is not rare in RVO, studies on RVO-induced SRH are limited and usually involve elderly patients. Zhao et al. suggested that SRH in RVO was related to damage to retinal circulation, and patients with SRH had a worse visual prognosis due to its toxic effect on the photoreceptor outer segments [21]. In newborns whose visual systems are rapidly developing, persistent RH will probably lead to amblyopia. In our case, SRH resolved in 3 weeks without ocular treatment, and OCT showed normal retinal structure at the last visit. The long-term influence of SRH on the retina and visual function still needs further research.

A limitation for this study is the absence of FA. FA for infants is usually performed under general anaesthesia. After consulting with a neonatologist, we decided to observe the fundus using only the RetCam system and indirect ophthalmoscopy based on the child’s poor systemic condition and the absence of clinically obvious neovascularization. The early OCT examination was also absent because the time spent was too long for this child to suffer in the initial 2 weeks. Another limitation is the short follow-up time. We could not analyse the long-term effects of stroke and RVO on the patient’s neurological and visual function.

This is the first report of a preterm neonate with simultaneous stroke and RVO. Although RVO is more likely to occur in people with a stroke history, it usually occurs a few years after stroke. Ophthalmologic fundus examinations are not routinely performed on neonatal stroke patients, and some of these patients may have experienced undetected retinal vascular diseases because they cannot express the sensation of a vision change and are easily overlooked. If retinal diseases persist or worsen, the visual prognosis will also deteriorate. A screening fundus examination for neonatal stroke patients seems to be meaningful; however, the feasibility and necessity still need further investigation.

## Data Availability

The datasets are available from the corresponding author on reasonable request.

## Abbreviations

RVO: Retinal vein occlusion
OCT: Optical coherence tomography
ME: Macular edema
FA: Fluorescein angiography
MRI: Magnetic resonance imaging
MRA: Magnetic resonance angiography
MCA: Middle cerebral artery
ICA: Internal carotid artery
RH: Retinal hemorrhage
AHT: Abusive head trauma
ICP: Intracranial pressure
DVT: Deep vein thrombosis
SRH: Subretinal hemorrhage
